# Impact of COVID-19 pandemic on the continuity of care for dermatologic patients on systemic therapy during the period of strict lockdown

**DOI:** 10.1016/j.amsu.2020.11.056

**Published:** 2020-11-25

**Authors:** Diala M. Alshiyab, Firas A. Al-qarqaz, Jihan M. Muhaidat

**Affiliations:** Department of Dermatology, Faculty of Medicine, Jordan University of Science and Technology, Irbid, Jordan

**Keywords:** COVID-19 pandemic, Corona virus, Skin diseases, Systemic treatments

## Abstract

**Background:**

The world has changed dramatically since the COVID-19 pandemic began. Jordan was among countries which enforced early lock-down for most non-vital services. Health care was mainly directed to cope with COVID-19 cases. The pandemic posed challenges for all patients, including dermatology patients especially those on systemic treatments. This resulted in interruption of medical care and exacerbation of pre-existing skin diseases for many patients.

**Material and methods:**

A cross-sectional, questionnaire-based study of dermatology patients on systemic treatment prior to corona pandemic. After lockdown was lifted, patients taking systemic treatments were evaluated for continuity of care during lockdown period and how that affected their skin condition. Demographic data, details of skin condition, continuity of care and impact on skin condition data were collected and analyzed.

**Results:**

154 patients (120 males, 34 females) were included. The majority (around 80%) of patients were unable to attend to dermatology clinics or do the needed lab monitoring. Around one fifth of patients had drug interruption mostly due to no access to hospital pharmacy. Most patients were using oral isotretinoin for acne, others include methotrexate and other immune suppressive agents. Patients with acne and oral isotretinoin treatment were more likely to continue their treatment during lockdown period. Amongst those who stopped treatment, around 42% had flare up of their disease.

**Conclusion:**

COVID-19 pandemic had an important impact on various aspects of care for dermatology patients especially those on systemic therapy. This study demonstrated limited access to specialist care, inability to do lab tests and discontinuation of treatment during lockdown. Some patients (42%) had flare up of their skin condition as a result.

## Highlights

• COVID-19 pandemic had dramatic impact on the continuity of care for dermatologic patients especially those on systemic therapy.
• Health authorities should ensure proper access to medical care for especially patients with severe skin disease treated with systemic drugs during periods of lockdown to minimize complications for these patients.

## 1 Introduction

The world has been dramatically affected by COVID-19 pandemic. Many aspects of life were greatly hammered but above all, health sector was stretched to the limit and beyond in many countries trying to cope with the huge number of COVID-19 cases [1,2]. The Jordanian government followed a strict lockdown approach to stop or slow number of new COVID 19 cases [3,4]. This approach had a severe impact on various aspects of daily life including shopping, all leisure activities, travel and importantly on medical sector among others. For a period of over 2 months (mid-march 2020 till first week of June 2020) the curfew affected all aspects of life for this population including a very limited access to medical care which was limited during this time to COVID 19 cases or emergency non-COVID cases. This has limited the medical care for patients with chronic non-life threatening illnesses including many dermatology patients. Although the government has tried to provide basic service via general practice clinics mainly for medication renewal this still did not provide the correct care for many of the patients who need specialist care, appropriate lab investigations or other interventions (e.g dose modifications … etc) which could not be provided by clinics without dermatology specialists.

The primary aim of this study is to assess continuity of medical care for dermatology patients during lock down (continuation of treatment, clinical follow up, lab investigations) especially patients with skin conditions treated with systemic medications prior to curfew. Additionally, to measure the impact of curfew on control of skin condition for these patients.

## 2 Materials and methods

### 2.1 Setting

A cross-sectional questionnaire-based study was conducted at our dermatology clinics at King Abdullah University Hospital (KAUH), Jordan University of science and Technology, Irbid, Jordan.

### 2.2 Study design

A cross sectional, questionnaire-based design that included patients on regular systemic medications (e.g Methotrexate, cyclosporine, oral retinoids and others) who were being followed in our outpatient dermatology. The questionnaire included demographic data regarding the (patients’ age, gender), clinical data related to skin disease, type of systemic treatment. Additionally, questions regarding the medical care if provided during lock-down, if the treatment was continued or interrupted, if the patient was able to do the needed follow up investigations, if was able to contact hospital or any medical center during lock-down, the impact of this period on the skin condition If treatment was stopped (Flare up, same or better).

### 2.3 Patients

Patients taking regular systemic treatment for their dermatologic conditions including methotrexate, cyclosporine, oral retinoids, azathioprine, mycophenolate mofetil, hydroxychloroquine and biological agents were included if the treatment was initiated prior to corona pandemic. Patients on oral antibiotics or on topical treatments only were excluded.

Patients who resumed follow up at dermatology clinics during the first month after lockdown were evaluated using the data questionnaire designed to collect information about the various aspects of medical care during the period of lockdown.

### 2.4 Statistical analysis

Statistical analysis was performed using the IBM SPSS software version 23. The median and inter-quartile range (IQR) is provided for quantitative variables. A p-value of <0.05 was considered statistically significant.

The study was approved by the local Institutional Review Board (IRB) committee (213/132/2020).

## 3 Results

A total of 154 patients were included (male n = 120, female n = 34). The median age was 25 years (6–72 years). Overall the majority of patients had interruption of their medical care from various aspects including: inability to attend dermatology clinics, not doing the required lab investigations and interruption of drugs administration. Table 1 shows the demographic and clinical data as well as continuity of medical care details during lockdown period.

**Table 1:** Demographic data and continuity of treatment.

Variable	Continued treatment					P-value
	No		Yes			
	N	%		N	%	
**Age**						
20 year or less	17	11.0%		52	33.8%	.288
21 year or more	15	9.7%		70	45.5%	
**Gender**						
Male	23	14.9%		97	63.0%	.354
Female	9	5.8%		25	16.2%	
**Diagnosis**						
Acne	20	13.0%		98	63.6%	.034
Others	12	7.8%		24	15.6%	
**Duration**						
<6months	16	10.4%		71	46.1%	.405
>6 months	16	10.4%		51	33.1%	
**Treatment**						
Isotretinoin	17	11.0%		91	59.1%	.018
Others	15	9.7%		31	20.1%	
**Able to come hospital**						
No	29	18.8%		95	61.7%	.105
Yes	3	1.9%		27	17.5%	
					
**Lab Investigations**						
No	29	18.8%		92	59.7%	.062
Yes	3	1.9%		30	19.5%	

124 patients (80.5%) were unable to come to hospital during lockdown and actually no out-patient clinics were available during that period. Since this lockdown was affecting public and private sectors (excluding emergency cases) only 13/124 patients tried to get specialist care in other private hospitals usually within the context of an emergency department with dermatologists being asked to see these patients in the ER. None of these patients had a life-threatening condition during that time.

**Fig. 1:**
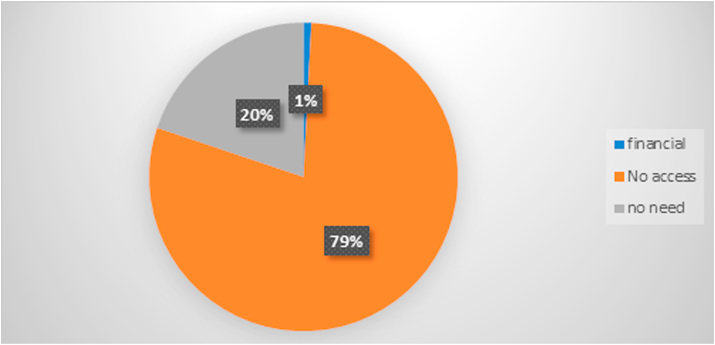
Reasons for not doing investigations.

78.6% of patients were unable to do the required follow up investigations related to their disease or the treatment. The main reason for not doing the needful lab tests was again having no access to hospital lab (79% of patients). Fig. 1 shows the main reasons for not doing the required labs during lockdown.

**Fig. 2:**
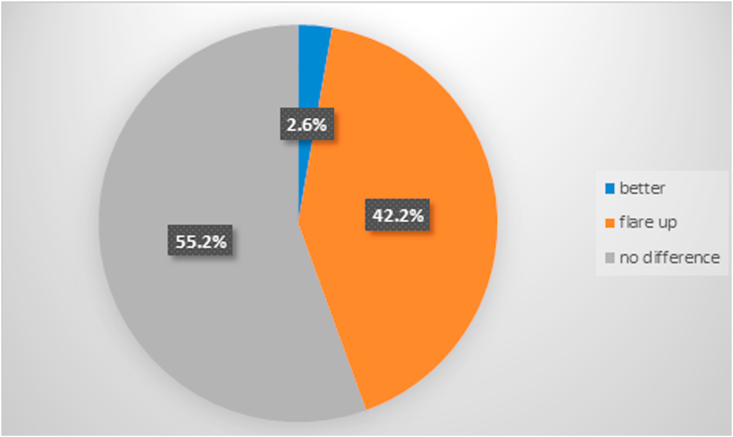
The impact of stopping treatment on skin condition.

Most patients (79.2%) were able to continue their systemic treatment during the lockdown period. The patients were able to get medications from hospital pharmacy through a delivery service or otherwise from outside pharmacies. Interruption of treatment (around 20.8% of patients) during lockdown ranged from 2 to 118 days with a mean of 62 days. The list of medications include oral isotretinoin (n = 119), Methotrextae (n = 20), cyclosporine (n = 4),mycophenolate mofetil (n = 4), Hydroxychloroquine (n = 4) and azathioprine (n = 2), Adalimumab (n = 1). The most common diagnoses for which these systemic drugs were used are: acne (most common 74.6%), psoriasis, atopic eczema, urticaria, discoid lupus, morphoea, pemphigus vulgaris and bullous pemphigoid. Patients with acne and treated by oral isotretinoin were more likely to have continuation of the treatment compared to other diagnoses (P = 0.03). On the other hand, other factors assessed like age, gender and durations of treatment (more or less than 6 months), whether lab investigations done or not, did not affect the continuity of treatment (p > 0.05). These results are shown in Table 1. The effect of treatment interruption on skin condition was associated with flare up in 65 patients (42.2%), while 85 patients (55.2%) showed no difference in their condition. interestingly, 4 patients (2.6%) noticed improvement in their skin conditions (Fig. 2). Flare up of skin condition was not related to diagnosis or duration of therapy. 45% of patients who had flare up tried a topical agent they had at home to control condition.

## 4 Discussion

COVID 19 had a dramatic impact on almost all aspects of life in a way we never experienced before. For the first time in our history, all non-vital activities came to almost a standstill. The health sector on the other hand was tested in many ways during this time. On the one side, many hospitals, and medical staff were stretched beyond limits dealing with large numbers of COVID cases. On the other hand, many medical services providing care to various other medical conditions was interrupted or affected directly or indirectly by COVID 19 pandemic [[5], [6], [7]]. The impact of this pandemic on patients with skin disease was demonstrated in several publications [[8], [9], [10]].

The results of this study clearly show that COVID 19 pandemic and subsequent lockdown had an important impact on patients with skin disease especially those with more severe diagnoses who require systemic treatment, frequent follow up and lab investigations. The lockdown prevented many patients from being able to come to dermatology clinics since access to medical care was restricted for suspected COVID 19 cases and emergency cases. Additionally, all out-patient services including public and private clinics were suspended during the lockdown. More than 80% of our patients were unable to get proper follow up from their specialists. Few patients managed to get a form of follow up through emergency departments. Another important aspect of care that was affected in these patients is the inability to do the required lab investigations needed for more than three quarters of these patients. Such lab tests are important either due to their condition or the systemic drug they were using. In some of these patients, being treated with immune suppressive therapy such investigations are important for the safe continuation of therapy as well as monitoring of side effects of these drugs.

The other important part of medical care for these patients includes providing regular treatment with the needed drug. This aspect was affected in around one fifth of patients. The hospital pharmacies adopted a policy of home drug delivery via volunteers (usually medical students) which helped many of these patients to get their drug supply. Also, private pharmacies were allowed to operate under certain conditions which also helped in providing alternative access to drugs apart from hospital pharmacy. Patients with acne were more likely to continue their treatment than patients with other diagnoses which could be explained by the fact these were younger patients, possibly more keen on continuing their treatment while other patients were generally older and more likely to stay at home during the lockdown period.

The reflection of this interruption of medical care was shown by flare ups of their skin condition seen in around 42% of patients. These flare ups could be the result of treatment interruption, lack of clinical follow which could result in improper dose adjustment, adding other lines of therapy … etc. the financial implications for these interruptions and flares are no doubt significant and should also be considered.

## 5 Conclusions

COVID-19 pandemic had dramatic impact on the continuity of care for dermatologic patients especially those on systemic therapy during the period of strict lockdown. This was shown by interruption of out-patient follow up, inability to do required lab monitoring investigations and discontinuation of systemic drugs resulting in suboptimal care and flare up of skin condition. Health authorities should ensure proper access to medical care for especially patients with severe skin disease treated with systemic drugs during periods of lockdown to minimize complications for these patients. Also this may be more cost effective than having to restart treatments or control severe disease flare ups.

## Study limitations

The number of patients is relatively small. a study with larger numbers and longer duration may be more confirmatory.

## Data availability

Data used in the article is available on request from the corresponding author.

## Declaration of competing interest

The authors declare no conflict of interest in relation to this study.

## Registration of Research Studies

Name of the registry: Impact of COVID-19 pandemic on the continuity of care for dermatologic patients on systemic therapy during the period of strict lockdown
Unique Identifying number or registration ID: researchregistry6048
Hyperlink to your specific registration (must be publicly accessible and will be checked): https://www.researchregistry.com/browse-the-registry#home/registrationdetails/5f6b99ed41d1dd00156d8576/
